# Determination of the Optimum Harvest Window for Apples Using the Non-Destructive Biospeckle Method

**DOI:** 10.3390/s16050661

**Published:** 2016-05-10

**Authors:** Anna Skic, Monika Szymańska-Chargot, Beata Kruk, Monika Chylińska, Piotr Mariusz Pieczywek, Andrzej Kurenda, Artur Zdunek, Krzysztof P. Rutkowski

**Affiliations:** 1 Faculty of Production Engineering, Department of Mechanical Engineering and Automatics, 28 Głęboka St 20-612 Lublin, Poland, University of Life Sciences, Akademicka 13, 20-950 Lublin, Poland; anna.skic@up.lublin.pl; 2 Institute of Agrophysics, Polish Academy of Sciences, Doswiadczalna 4, 20-290 Lublin 27, Poland; m.szymanska@ipan.lublin.pl (M.S.-C.); b.kruk@ipan.lublin.pl (B.K.); m.chylinska@ipan.lublin.pl (M.C.); p.pieczywek@ipan.lublin.pl (P.M.P.); a.kurenda@ipan.lublin.pl (A.K.); 3 Research Institute of Horticulture, Pomologiczna 18, 96-100 Skierniewice, Poland; Krzysztof.Rutkowski@inhort.pl

**Keywords:** biospeckle, apple, maturity index, Streif, optimum harvest window, postharvest quality

## Abstract

Determination of the optimum harvest window plays a key role in the agro-food chain as the quality of fruit depends on the right harvesting time and appropriate storage conditions during the postharvest period. Usually, indices based on destructive measurements are used for this purpose, like the De Jager Index (PFW-1), FARS index and the most popular Streif Index. In this study, we proposed a biospeckle method for the evaluation of the optimum harvest window (OHW) of the “Ligol” and “Szampion” apple cultivars. The experiment involved eight different maturity stages, of which four were followed by long cold storage and shelf life to assist the determination of the optimum harvest window. The biospeckle activity was studied in relation to standard quality attributes (firmness, acidity, starch, soluble solids content, Streif Index) and physiological parameters (respiration and ethylene emission) of both apple cultivars. Changes of biospeckle activity (BA) over time showed moderate relationships with biochemical changes during apple maturation and ripening. The harvest date suggested by the Streif Index and postharvest quality indicators matched with characteristic decrease in BA. The ability of biospeckle method to characterize the biological state of apples was confirmed by significant correlations of BA with firmness, starch index, total soluble solids and Streif Index, as well as good match with changes in carbon dioxide and ethylene emission. However, it should be noted that correlations between variables changing over time are not as meaningful as independent observations. Also, it is a well-known property of the Pearson’s correlation that its value is highly susceptible to outlier data. Due to its non-selective nature the BA reflected only the current biological state of the fruit and could be affected by many other factors. The investigations showed that the optimum harvest window for apples was indicated by the characteristic drop of BA during pre-harvest development. Despite this, at the current state of development the BA method cannot be used as an indicator alone. Due to rather poor results for prediction in OHW the BA measurements should be supported by other destructive methods to compensate its low selectivity.

## 1. Introduction

Determination of the optimum harvest window plays a key role in the agro-food chain as the quality of fruit depends on the right harvesting time and appropriate storage conditions during the postharvest period [1,2,3,4,5,6]. To ensure the highest quality at the end of long-term storage, apples must be harvested when mature but not yet fully ripe [7,8]. When apples are harvested immaturely they do not develop their full ripeness after storage, which leads to a small size, poor fruit colour, sour and starchy flavour and a weak aroma. They are also more susceptible to scald, bitter-pit and internal breakdown. Mass reduction by water loss is also greater in apples picked earlier because the cuticle is not completely formed at this moment [9,10]. Conversely, apples harvested when over-mature are vulnerable to mechanical injury and disease, and sensitive to low temperature breakdown or watercore [11]. All these physiological processes take place even under optimum conditions, which complicates storage [12,13]. Hence, determination of the optimum harvest date (OHD) is a crucial issue for growers to ensure the high post-storage apple quality expected by consumers.

Apple harvest at the pre-climacteric minimum, when the respiratory activity is minimal, is considered as optimum, because the storage potential remains high and the organoleptic quality is near its maximum. Many methods of fruit maturity stage evaluation have already been developed and can be divided into destructive and non-destructive methods. Despite being time consuming and expensive, destructive methodologies have been traditionally used as reference measurements of fruit quality and involve standard chemical analysis based on the evaluation of starch content, soluble solid content, titratable acidity or measurement of firmness. Prediction of OHD is possible by direct measurement of these quality attributes some weeks before the estimated harvest date and calculation of the maturity index. The Streif Index [6], including measurements of firmness, soluble solid concentration and starch degradation index, has been successfully used to estimate the optimum harvest date [5,7,14]. The soluble solids and the starch index increase, while the firmness decreases in maturing fruit. As a result, the Streif Index declines during fruit development—starting from five or six and reaching values between 0.30 and 0.08 at the OHD [15]. The index fluctuations, if observed in the period starting a dozen days before harvest, allow the optimum harvest date to be determined a few days in advance [16]. In addition, alternative criteria like the De Jager Index (PFW-1) [17] and the FARS Index [5,7] may be used for harvest date determination, but they have never been used commercially.

Various non-destructive systems have been developed for evaluation of the fruit quality. Among them optical methods like VIS/NIR spectroscopy [18,19], time-resolved reflectance spectroscopy [9], hyperspectral backscattering imaging [20,21], laser induced backscattering [22,23] or chlorophyll fluorescence [24,25,26] are the most promising. Dielectric spectroscopy techniques were also successfully used for examination of the properties of agricultural materials [27]. Non-destructive methods are fast and robust, allowing one to analyse a large number of samples. Moreover, it is possible to carry out continuous measurements on the same samples during different stages of maturation as well as in the post-harvest period. Such methods also provide commercial tools for classifying fruit and quality control and also may be used directly in the orchard.

The biospeckle method is another non-invasive technique based on the optical phenomenon which occurs during the illumination of a samples’ surface by coherent light such as that produced by a laser [28]. The wavefronts of scattered rays interfere with each other and form random, granular patterns consisting of dark and bright spots, visible on the observation plane. The diffraction pattern depends on the geometry of the system, the wavelength of the laser and the aperture of lens of capturing device. The speckle pattern composed of dark and light spots is static for non-living matter, but in the case of biological samples, the intensity distribution pattern evolves and fluctuates over time [29,30]. Draijer *et al.* suggested that this dynamic behavior is mainly caused by Doppler shifts of the light as it interacts with moving particles [31]. According to [32] processes such as cytoplasmic streaming, organelle movement, cell growth and division during fruit maturation and biochemical reactions are responsible for certain biospeckle activity. Besides those, the biospeckle fluctuations depend on the chlorophyll [33] and starch content [34], occurrence of fungal infections [35], and temperature of the investigated plant samples [36].

So far, applications of the biospeckle technique in the agriculture area include determination of the quality and maturation degree of fruits and vegetables [37,38,39,40,41], analysis of seeds [42,43] or detection of plant root bioactivity changes [32]. In all cases biospeckle activity changed with the state of the investigated samples.

Information presented by other authors and conclusions drawn from our previous experiments suggested that the method could possibly be used for the determination of the optimum harvest date. This study is an extension towards the prediction of the optimum harvest date using the biospeckle method. Therefore, the goal of this study was to compare the OHD based on BA measurement as a non-destructive index, with other OHD calculated based on destructive analyses and physiological parameters commonly used in commercial orchards. Evaluation of OHDs selected with different methods was made by monitoring of the postharvest quality of the fruit during long term cold storage and shelf-life.

## 2. Materials and Methods

Two apple cultivars (*Malus Domestica*, cv. “Ligol” and “Szampion”) with distinct textural parameters were used in this study. The orchard was located in the Grojec region in Poland. Apples were collected during the period from 3 July (185th day of the year) to 4 October (278th day of the year). The majority of the period involved fruit development processes because the presumed optimum harvest window was expected to be between the 20 and 25 of September. In total, eight sampling dates which covered different maturity stages of fruit were considered in this experiment (Table 1). Fruits were collected from five trees for each cultivar. Sampling from trees was random. Fifteen fruits of similar size and maturity were collected on each picking date. Biospeckle activity (BA) and firmness (F) were measured for individual apples. Five halved apples were used for starch index (SI) determination. The remaining fruits were cored and cut into small portions, homogenized and used for the standard tests for maturity state evaluation, such as starch content (SC), titratable acidity (TA) and soluble solid content (SSC). Ethylene and carbon dioxide emissions were measured starting from the 209th day of year (2 sampling date–27 July) and internal ethylene concentration was determined from the 226th day of year (3 sampling date–13 August), due to the absence of a well-developed ovary.

Moreover, additional apples picked between the 254 and 278 days of the year (5th–8th sampling dates) were stored for one, two and three months under a normal atmosphere, at 2 °C. After the storage apples were tested at one and seven days of shelf life at room temperature. Firmness, soluble solids, starch content, titratable acidity and biospeckle activity were measured for these fruits.

### 2.1. Biospeckle Activity (BA)

**Figure 1:**
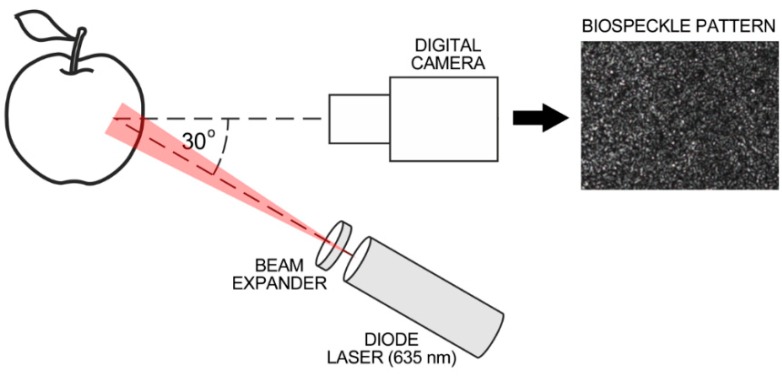
Schematic illustration of biospeckle measurement system.

The system used for biospeckle measurement was as presented previously [28,33,35,36] and is illustrated schematically in Figure 1. It consisted of a diode laser, (8 mW, λ = 635 nm, LQC635-08C, supplied with a Laser Diode Control Unit, Newport, Irvine, CA, USA), a laser beam expander 20× (Edmund Optics GmbH, Karlsruhe, Germany) and a CCD camera (Monochrome FireWire Astronomy Camera DMK 21AF04.AS, The Imaging Source Europe GmbH, Bremen, Germany) with a 1:14 25 mm TV lens and 20 mm extension ring (Pentax Corporation, Tokyo, Japan).

The distance between the camera and the apple was about 85 mm and between the laser and the apple the distance was 180 mm. The illumination angle was θ ≈ 30°. The image exposure time of the CCD camera was 1/250; gain and brightness were set experimentally to avoid pixel overexposure on the image histogram. The image resolution was 320 × 240 pixels that corresponded to an observation area of about 3 × 2 mm. The relatively small field of view reduced the influence of apples’ curvature on the distribution of illumination intensity. The image acquisition parameters were kept unchanged during the experiments.

Biospeckle phenomena were recorded as four second-long video sequences (uncompressed AVI) captured with a rate of 15 fps. Based on our previous studies biospeckle activity was evaluated using the correlation coefficient C calculated between the first and the last frame of video sequence [33,34,35,36,37]. The C coefficient was calculated using the standard in-build functions in Matlab^®^ R2010a software (MathWorks, Natick, MA, USA). Finally, the biospeckle activity was expressed as BA = 1 − C. Each apple was tested at two locations on the fruit equator on opposite sides and then the mean value of BA for the fruit was used.

### 2.2. Firmness (F)

Apple firmness (F) was measured using a universal testing machine Lloyd LRX (Lloyd Instruments Ltd., Hampshire, UK) with a 500 N load cell. Apple firmness was determined by means of a standard puncture test using a cylindrical probe (11.1 mm diameter) at a speed of 20 mm/min. Before the test the apple skin was cut off. The maximum force needed to penetrate the flesh over a distance of 8 mm was read as the apple firmness (F) and expressed in N.

### 2.3. Soluble Solid Content (SSC)

Total soluble solid content (SSC) was determined using a digital refractometer (PAL-BX/R1, Atago Co. Ltd., Tokyo, Japan). Apple pulp was filtered and then a drop of filtrate was placed onto a prism of the refractometer. Soluble solid content was measured using the Brix scale. The measurement was performed five times for each sample.

### 2.4. Titratable Acidity (TA)

Approximately 40 g of each apple pulp was weighted in triplicate with 0.001 g accuracy. Pulp was placed in a volumetric flask and 100 mL of deionized water was added. The suspension was heated to boiling point and then cooled to room temperature. Deionized water was added up to 250 mL. After that, the pulp suspension was filtrated and the water extract (50 mL) was titrated to pH 8.1 with 0.1 M NaOH. Measurements were conducted three times for each filtrate. Results were calculated as malic acid and expressed as g/100 g apple fresh weight.

### 2.5. Starch Index (SI)

In order to evaluate the starch index (SI) five apples were cut in half across the equator, dipped for one minute in an iodine solution (40 g KI + 10 g I_2_ in 1 L H_2_O) and dried. The color patterns that appeared after the iodine treatment were compared with color reference charts, which rated SI using one (all stained) to ten (not stained) Eurofru scale (Ctifl , France). Results were expressed as mean values of two independent ratings.

### 2.6. Starch Content (SC)

The evaluation of the starch content (SC) in the apples was based on the Megazyme Total Starch Assay Kit (Megazyme Inc., Chicago, IL, USA), which is suitable for the measurement of total starch in most native and processed cereal products, as well as other plant materials. Hydrolytic conversion of starch to glucose was performed in two phases. In the first phase, starch, water and thermostable α-amylase were mixed and heated to boiling point. The starch was totally solubilized and became partially hydrolysed to dextrin. In the second phase, the resulting starch dextrins were quantitatively hydrolysed to glucose by amyloglucosidase. Finally, the glucose oxidase/peroxidase reagent was added to samples and absorbance against a reagent blank was read at around 510 nm. The measurement was performed three times for each sample.

### 2.7. Ethylene and Carbon Dioxide Emission

For internal ethylene concentration (IEC) a 1 mL gas sample was taken from the apple core and injected into a HP 5890 II gas chromatograph (Hewlett-Packard Inc., Palo Alto, CA) equipped with an alumina packed glass column. Results were expressed in µL/L (ppm). Moreover, in order to measure the production rates of carbon dioxide and ethylene, apples were placed individually in 1.8 L glass jars equipped with septa. The jars were hermetically closed for two hours and then two samples of 1 mL each were taken (one for ethylene and the second for carbon dioxide). The carbon dioxide was measured using an ADC 225 MK3 gas analyser (ADC Gas Analysis Ltd., Hoddesdon, UK). Results were expressed in μL·kg^−1^·h^−1^ (ethylene production rate) and μL·kg^−1^·h^−1^ (carbon dioxide production).

### 2.8. Maturity Index

Streif Index combines three parameters, which change quickly during the preharvest period, according to following equation:

(1) $$\mathrm{Streif index}=\frac{\text{F}}{\mathrm{SSC}\cdot \mathrm{SI}}$$

where F (firmness) is expressed in kg, SSC is the soluble solid content and SI is the starch index. Streif Index, if observed starting a dozen days before harvest, allows to determine the optimum harvest date a few days in advance [16].

### 2.9. Statistical Analysis

Statistical analysis was carried out using the commercial Statistica 10.0 (StatSoft, Inc., Tulsa, OK, USA) software. Analysis included calculations of average values and standard deviations for all measured parameters, as well as significance of their changes over time, investigated by means of one-way ANOVA test (using *post hoc* Tukey’s Honestly Significant Difference—HSD) for each cultivar independently. The relationship between biospeckle activity (BA) and other maturity indices was analyzed using Pearson’s correlation coefficients (R). Additionally, the effect of month of storage*shelf life day was investigated using two-way ANOVA test for stored apples.

## 3. Results

### 3.1. Preharvest Monitoring for the Optimum Harvest Date

**Figure 2:**
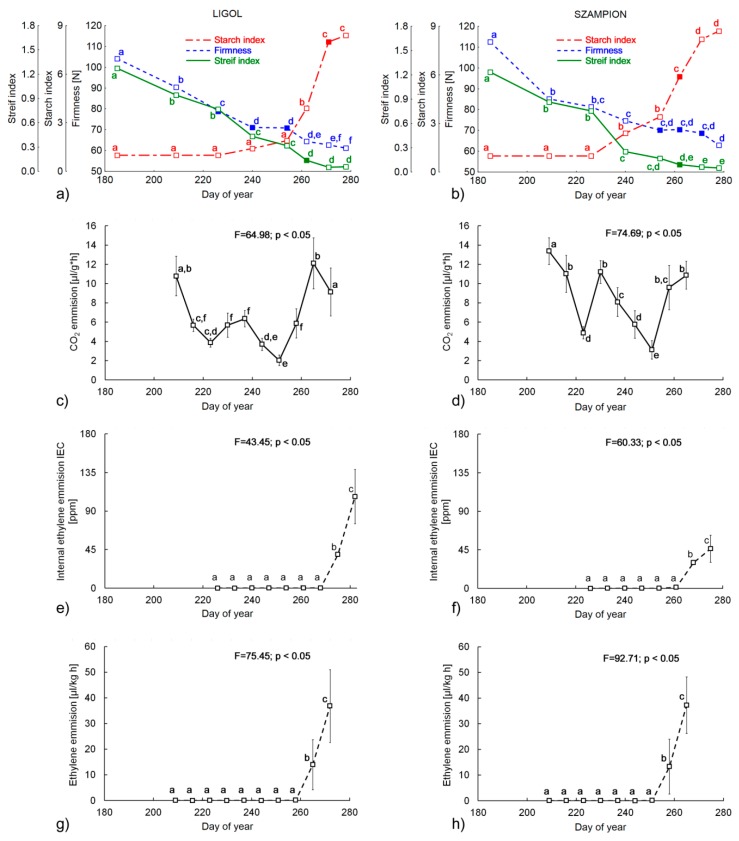
The changes of (**a**,**b**) Streif Index, Starch Index and Firmness (**c**,**d**) carbon dioxide emission, (**e**,**f**) internal ethylene concentration (IEC) and (**g**,**h**) the rate of ethylene production for ‘Ligol’ (on the left) and ‘Szampion’ cultivars (on the right side). The filled markers indicate optimum harvest date found. Bars represent standard deviations. The superscript letters denote no significant differences at *p* < 0.05.

The results of the firmness and chemical analyses regarding picking dates are presented in Table 1 and Figure 2. As expected, firmness for both cultivars decreased significantly during the studied period (Figure 2a,b), which is in good agreement with results obtained in previous studies [8,37]. It is commonly accepted that optimal firmness at harvesting date for “Szampion” apples should be in the range of 68–73 N, whereas for “Ligol” it should be 75–80 N. Taking only firmness into consideration, the harvest date should be selected between the 226th and 254th day of the year (3rd–5th sampling dates) for “Ligol” and between the 254th and 271st day (5th–7th sampling dates) for “Szampion”. Titratable Acidity (TA) decreased gradually during the experiment. Previous studies showed that the decreasing tendency of TA should be typically expected during fruit development, although there are also reports that TA could strongly vary between seasons. Determination of titratable acidity is rarely used in commercial practice, although it is usually performed in research projects evaluating storage conditions [8,44].

Starch content (SC) initially remained constant during the fruits’ development and diminished on the last three sampling dates, showing in total a significant decrease for both cultivars. This result is consistent with previous investigations [37,45]. The most pronounced increase was observed for total soluble solids SSC (F-value > 1300) for both tested cultivars, which was connected with starch degradation. Starch index (SI) is also an important indicator of apple maturity state. It increases as the starch content decreases and is a result of starch granule hydrolysis into simpler carbohydrates during apple maturation. “Ligol” apples should be harvested when the starch index is between six and eight and for “Szampion” SI should be in the range of five to seven. Based on these values, the optimum harvest date should be at the 262nd and 271st day (6th and 7th sampling dates) in the case of “Ligol” and “Szampion” apples, respectively. It is clear that harvest windows obtained with all measured parameters separately do not match with each other, which leads to conclusion that complex measures should be used for this purpose. SSC and TA are also considered to be important factors for consumers [3,44]. However, due to the fact that the acid level at harvest varies greatly between seasons and growing regions, TA is considered to be less reliable indicator of the harvest maturity [46,47]. The Streif Index, which combines firmness, starch index and total soluble solid content, is commonly used in horticultural practice [16]. The variations of this index during fruit development (Figure 2a,b) were similar to that obtained in the literature—an initial rapid decrease and then a small variation near the optimum harvest date [5,7,14]. In our experiment the values of the Streif Index dropped from 1.26 to 0.06 for “Ligol” and from 1.23 to 0.05 for “Szampion” (Figure 2a,b). Thus, the optimum harvest data in terms of the Streif Index should be on the 262nd day of year (6th sampling dates) for both tested cultivars (indicated by filled markers on Figure 2a,b).

The rate of respiration processes serves as an indicator of the fruit maturation degree and the physiological state of the tissues [48]. During the growing season, apple respiration gradually declines until it reaches a minimum several weeks before the fruit ripens. This is known as the pre-climacteric minimum. Apple maturation is also characterized by a large increase in ethylene production, which accompanies the respiratory peak during ripening, called the climacteric crisis [49].

The rate of carbon dioxide and ethylene emissions was measured starting from the 209th day of the year (2nd sampling date, 26 July) at weekly intervals, to observe the characteristic increase in respiration rate and ethylene synthesis. The CO_2_ emission change profiles were similar for both cultivars. Initially, the rate of carbon dioxide emissions decreased by the 223rd day of year (4th sampling date). After that, between 230th and 237th day (Figure 2c,d) there was a clear increase and then a continuous decrease up to the 244th day of the year. After that, a rapid increase in respiration rate occurred for both cultivars (Figure 2a,b).

Ethylene emissions were constant from the 209th to the 258th and from the 209th to the 251st day for “Ligol” and “Szampion”, respectively. After that, a significant increase was noted (Figure 2g,h). The internal ethylene concentration (IEC) was also measured in the experiment, starting from the 226th day of the year (3rd sampling date–13 August). The profile of the IEC changes (Figure 2e,f) was similar to the ethylene emission profile (Figure 2g,h). Initially, the internal ethylene concentration remained constant (close to 0 ppm) and after the 268th day of the year it increased significantly. Taking into account changes in the respiration and ethylene biosynthesis rate it should be pointed out that apples of both tested cultivar were at their pre-climacteric phase from the 237th to the 261st day. This indicates that fruits should be harvested at the 261st day of the year at the latest.

### 3.2. Postharvest Quality of Apples

In order to check which harvest date was optimum for long-term storage, an experiment combining cold storage with shelf life was a conducted for apples harvested at sampling dates ranging from five to eight. Three storage months as well as one and seven shelf life days were taken into account in the statistical analysis. The performed ANOVA showed that the effect of month of storage×shelf life day on quality attributes was significant in most cases for both tested cultivars (Table 2). As expected, SI increased during storage, which means that starch degradation still occurred. TA and SSC did not change much during storage although a slight increase was observed. Firmness decreased during cold storage for both cultivars in all developmental stages. The lowest decrease in firmness was reported for apples harvested at the 262nd day of the year (6th developmental stage) for both tested cultivars (Table 2). Thus, it could be concluded that the 262nd day of the year (6th sampling date) was the optimum for both cultivars from the point of view of postharvest storability in a cold room.

### 3.3. Biospeckle Activity

**Figure 3:**
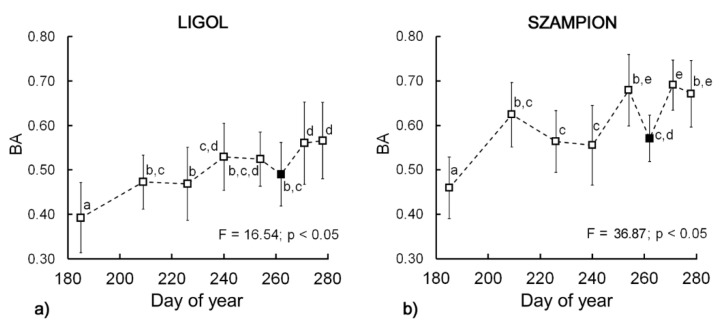
Biospeckle activity changes during (**a**) “Ligol”; and (**b**) “Szampion” apple development and maturation. Bars represent standard deviations. The superscript letters denote no significant differences at *p* < 0.05. The filled square marker indicates optimum harvest date.

Biospeckle activity showed an overall significant increase (*p* < 0.05) during the experiment (Figure 3a,b) for both cultivars. For “Ligol” the total changes were less pronounced (F = 16.54) and more regular, whereas for ‘Szampion’ the total increase was much larger (F = 36.87), however some discrepancies from the ascending trend appeared. The ascending trend of BA values was in good agreement with previous studies using the biospeckle method for preharvest apple monitoring [37]. Figure 3 shows that at the 262nd day (6th sampling date) biospeckle activity decreased for both tested cultivars and then increased again.

Biospeckle activity was also measured during cold storage. However, the trends varied depending on the harvest date. For apples picked at the 254th day (5th sampling date) BA increased during cold storage, whereas at the 271st and 278th day (7th and 8th sampling date) BA decreased (Table 2). Apples harvested on the 262nd day (6th sampling date–18th of September) were characterized by the lowest fluctuation of BA values during cold storage.

## 4. Discussion

Harvest date has a great impact on maintaining firmness during the storage. Apples harvested at the optimal harvest date usually tend to lose their firmness much slower during the long term storage. Thus, the stage of maturity at harvest directly affects quality of the product. The lowest decrease in firmness was reported for apples picked on the 18th of September (6th sampling date) both after storage for three months and during the shelf life. This suggested that apples harvested at 262nd day (6th sampling date) had the best storage capabilities and were suitable for delivery to the market. Also, fruits picked up at 6th sampling date showed the smallest changes of BA after harvest. Considering the above, for “Ligol” and “Szampion” apples intended for long cold storage the optimal harvest date was at the 18th September during the analyzed growth season. Fruit ontogenesis consists of maturation and ripening phases. The first one includes physiological, biochemical and morphological changes, and ends when the maximum size of the fruit is reached. Ripening involves structural and chemical modifications of fruit composition, resulting from processes such as the transformation of chloroplasts into chromoplasts, loss of chlorophyll and accumulation of carotenoids, softening of the tissue and changes in acids and sugars metabolism [50,51]. Fruit growth is maintained by the expansion of parenchyma cells, which in turn triggers processes in the cell and cell wall. Some photosynthetic products synthesized in leaves are accumulated in fruit parenchyma in the form of sugars. This accumulation plays a vital role in cellular water retention and sustains cell turgor. In addition, cell wall flexibility is crucial for cellular integrity and fruit expansion [52].

According to the literature [15] the optimum harvest time for apples occurs when the Streif Index is in range of 0.08–0.30. Particularly for “Szampion”, the Streif Index at harvest was found to be 0.12 [16]. Thus, the optimum harvest window in terms of the Streif Index for apples in this experiment should be on the 262nd day (6th sampling date) for both tested cultivars (Figure 2a,b).

During the harvest period, climacteric fruits, such as apples, show typical biochemical changes, which may influence the biospeckle activity. It already has been shown that for apples the BA is affected by changes in starch [34] and chlorophyll content [33]. Taking into account previous results [37], this experiment unambiguously showed that BA increases during fruit development, up to a small drop down that occurs just at the harvest time. It is possible that the observed drop down shown on the BA graph (Figure 3a,b) is a characteristic point that indicates the optimal harvest window. This observation was supported by comparison with the Streif Index, ethylene and carbon dioxide emission and postharvest firmness of fruits (Figure 2). At the very beginning of fruit development the initial increase of BA can be related to the first phase of ontogenesis, which involves cell division and enlargement. This stage of fruit development was also associated the highest intensity of respiration (Figure 2c,d). Following this stage, the growth of fruit was inhibited and ripening began. The respiration rate showed a descending trend during this phase, up to the 223rd day of the year. The lowest rate of carbon dioxide emission occurred at the 251st day. After that, an increase in respiration was observed, which is related to climacteric peak. This was confirmed by the increase in ethylene synthesis (Figure 2g,h) and internal ethylene concentration (Figure 2e,f). Considering the fact, that apple harvest at the preclimacteric minimum is regarded to be the optimal date, in our case, apples should be harvested no later than on the 261st day.

After storage for one, two and three months, changes in biospeckle activity as well as firmness were the lowest for apples picked on the 18th of September (6th sampling date). This gives additional evidence that apples harvested just after the occurrence of a decrease in BA had the best capabilities for retaining satisfactory quality after long-term storage. Table 3 shows the correlation matrix between BA and the traditional destructive indices used for the prediction of the optimum harvest date. To obtain these correlation data, BA, firmness and SI for individual fruits were taken, whereas SSC was measured from pulp. Correlation values varying from 0.4 to 0.63 for BA (Table 3), were considered as significant (on basis of the statistical analysis), but very weak correlations. Significant correlations between BA parameters and destructive maturity indices show that the biospeckle technique could be used as one of the methods for non-destructive characterization and prediction of the optimum harvest window for apples. However, it should be noted that it is possible that correlations of some of the variables are not a result of direct relationship, but rather similar underlying process causing changes over time. It should be noted that correlations between variables changing over time are not as meaningful as independent observations. Also, it is a well-known property of Pearsons correlation that its value is highly affected by outlier data.

The optimal harvest window for the tested cultivars was determined by grower as between the 20th and 27th of September, based on the maturity indices such as firmness, starch degradation pattern, background color change and intensity of blush. This corresponds to the period between the 262nd and 271st day of year (6th and 7th sampling dates), which matches with the occurrence of BA drop down.

Up to now apple maturity stage has been evaluated and predicted by many methods, starting from such sophisticated ones as the internal concentration of ethylene, moving to simpler methods, like the evaluation of firmness or concentration of starch. However none of the available measures is able to provide full characterization of biological stage of fruit alone. Therefore, attempts were made to create measures (De Jager index, FARS index, *etc.*) that combine multiple descriptors (firmness, starch content or even the coloration of fruit skin) condensed into one value, providing convenient indicators of maturity. Despite this efforts prediction of OHD still involves destructive methods and lacks precision. For instance, De Jager index calculated in this study did not show any extremum during studied period, which hinder the prediction of OHD.

The BA changes over time showed a clear drop near OHD. Due to its non-selective nature the biospeckle method reflects the physiological state of the whole fruit and can be affected by many other factors. The investigations showed that the optimum harvest window for apples was indicated by the characteristic drop of BA during pre-harvest development. However, at the current state of development the BA method cannot be used as an indicator alone. The rather poor performance suggests that BA measurements should be supported by other destructive methods to compensate for the low selectivity of this method. The great advantage of this method is its non-destructive nature in relation to the other methods used in this experiment. Unfortunately, the biospeckle method is highly sensitive to vibrations; its eventual application for evaluation of the optimum harvest window requires fruit to be picked from the tree and measured under stable conditions (indoor or outdoor but protected from vibration). The error of BA measurements also depends on the positioning of fruit against the laser and camera; however this is much less problematic than the typical large biological variability among fruits. This means that, according to our experience, the method requires BA measurement from batches of about ten apples and two to four positions on each apple, most often from the equator. However taking into account a four second measuring time, a typical skilled person can complete the test in about ten minutes.

On the other hand, new biospeckle setup arrangements may reduce the negative effects of sample vibrations as well as specular and directly reflected portion of light. It is possible to reduce the acquisition time by employing high speed cameras. Shorter acquisition times also result in reduced vulnerability to vibrations. High power light sources could be used to illuminate samples from the back side, thus only the transmitted portion of light would be captured.

Improvements can also be achieved in biospeckle data processing techniques. Recently, novel methods of analysis of speckle time-varying patterns based on frequency analysis were introduced. Frequency analysis allows the mapping of activities associated with specific biological or physical processes that only occurs at certain frequencies in the sample. Currently available applications employ direct analysis of frequency bands by means of statistical descriptors like mean amplitude value of harmonic frequencies or signal entropy *etc.* [31,53,54].

## 5. Conclusions

Apples harvested at the 6th picking date showed the best storage capabilities, which was indicated by the smallest decrease in firmness. This picking date was predicted as optimal both by means of Streif Index and BA analysis. The optimum harvest window was indicated by BA analysis as a characteristic decrease in measured activity. Moreover, a significant correlation between biospeckle activity and Streif Index was obtained, suggesting that changes of both measures were driven by similar processes, undergoing during fruit development and causing biophysical changes of fruit.

A significant correlation between biospeckle activity, firmness, starch index, Streif Index and total soluble solids was obtained, showing that monitoring BA during the growth season could be used with other maturity indices for optimum harvest window determination. Correlation values varying from 0.4 to 0.63, were considered as significant (on the basis of the statistical analysis), but very weak correlations. It should be emphasized that BA is not a selective method, which means that BA reflects only the current biological state of the fruit and can be affected by many other factors. Therefore, the BA measurements should be supported by other destructive methods to compensate its low selectivity. The biggest advantage of the proposed methodology is its non-destructive character and relatively low cost in comparison to destructive and time consuming chemical analysis. This approach can be considered as a fast alternative method allowing producers to improve fruit harvesting and storage.

## Figures and Tables

**Table 1:** Characteristics of apple maturity states expressed as the average values of measured parameters. In parenthesis standard deviation.

Development Stage		1	2	3	4	5	6	7	8	F-Value
Sampling date		3 July	27 July	13 August	27 August	10 September	18 September	27 September	4 October	
Day of year		185	209	226	240	254	262	271	278	
**Cultivar Ligol**	Starch content (mg/100 g dry weight)	13.16 (±0.53) ^a^	11.58 (±0.70) ^a,b^	10.36 (±1.14) ^a,b^	8.58 (±0.26) ^b,c^	8.07 (±0.46) ^b,c^	5.72 (±3.02) ^c,d^	2.46 (±1.10) ^d,e^	1.88 (±0.39) ^e^	31.58 *
	Titratable acidity (g/100 g of fresh weight)	0.542 (±0.010) ^a^	0.379 (±0.032) ^b^	0.332 (±0.016) ^c^	0.301 (±0.014) ^d^	0.278 (±0.002) ^d^	0.230 (±0.004) ^e^	0.226 (±0.004) ^e^	0.218 (±0.002) ^e^	176.74 *
	Total soluble solids content (°Brix)	8.34 (±0.05) ^a^	9.78 (±0.04) ^b^	10.52 (±0.11) ^c^	11.94 (±0.05) ^d^	12.00 (±0.04) ^e^	12.40 (±0.00) ^f^	15.54 (±0.05) ^g^	13.18 (±0.04) ^h^	4013.20 *
**Cultivar Szampion**	Starch content (mg/100 g fresh weight)	8.78 (±0.70) ^a,c^	11.56 (±0.68) ^b^	8.29 (±0.83) ^a,c,d^	7.58 (±0.05) ^a,c,d^	10.22 (±0.69) ^b,c^	5.55 (±2.29) ^d,e^	6.36 (±0.41) ^a,d^	2.97 (±0.27) ^e^	23.23 *
	Titratable acidity (g/100 g of fresh weight)	0.362 (±0.001) ^a^	0.269 (±0.013) ^b^	0.186 (±0.004) ^c^	0.186 (±0.012) ^c^	0.189 (±0.004) ^c^	0.127 (±0.001) ^d^	0.134 (±0.004) ^d^	0.138 (±0.001) ^d^	406.70 *
	Total soluble solids content (°Brix)	9.30 (±0.16) ^a^	10.04 (±0.05) ^b^	11.00 (±0.10) ^c^	12.54 (±0.15) ^d^	12.50 (±0.07) ^d^	13.30 (±0.10) ^e^	13.68 (±0.15) ^f^	14.66 (±0.09) ^g^	1300.90 *

^a, b, c, d, e, f, g, h^ Letter indicate membership to homogeneous groups (according to ANOVA with *p* = 0.05). The same superscript letter denotes no significant differences; * means the effect is significant at *p* < 0.05.

**Table 2:** Firmness and biospeckle activity of cv. “Ligol” and “Szampion” after one, two and three months of storage evaluated at the 1st and 7th day of shelf life. In parenthesis standard deviation. F-value of two-way ANOVA of month of storage*shelf life day effect.

	No. of Sampling Date			5		6		7		8	
	Sampling Date			10 September	F-Value	18 September	F-value	27 September	F-value	4 October	F-Value
		Month of Storage	Shelf Life Day								
**LIGOL**	**Firmness (N)**	1	1	65.93 (±3.03) ^a^	1.43	68.76 (±6.48) ^a^	3.45 *	69.20 (±8.58) ^a^	4.22 *	66.18 (±5.29) ^a^	3.85 *
			7	58.27 (±5.73) ^b^		57.48 (±5.83) ^b,c^		59.69 (±8.27) ^a,b^		58.61 (±9.22) ^a,b^	
		2	1	57.37 (±5.52) ^b,c^		61.85 (±8.82) ^a,b^		51.75 (±7.23) ^b,c^		67.18 (±12.49) ^a^	
			7	50.57 (±6.23) ^c,d^		52.60 (±10.74) ^b,c^		55.75 (±11.45) ^b^		48.21 (±5.67) ^b,c^	
		3	1	49.81 (±6.23) ^d^		51.28 (±7.89) ^c^		42.87 (±4.85) ^c^		50.04 (±13.20) ^b,c^	
			7	47.71 (±8.77) ^d^		51.84 (±9.30) ^c^		42.29 (±7.28) ^c^		44.73 (±5.17) ^c^	
	**Biospeckle activity BA**	1	1	0.475 (±0.065) ^a^	5.90 *	0.529 (±0.049) ^a^	5.30 *	0.580 (±0.058) ^b^	9.09 *	0.607 (±0.074) ^a^	22.39 *
			7	0.500 (±0.064) ^a,b,c^		0.562 (±0.049) ^a,b^		0.473 (±0.058) ^a^		0.457 (±0.076) ^b^	
		2	1	0.526 (±0.088) ^a,b,c^		0.566 (±0.057) ^b^		0.528 (±0.072) ^b^		0.530 (±0.062) ^c^	
			7	0.515 (±0.064) ^c^		0.535 (±0.064) ^a,b^		0.523 (±0.096) ^a,b^		0.534 (±0.059) ^c^	
		3	1	0.539 (±0.093) ^b,c^		0.550 (±0.056) ^a,b^		0.497 (±0.077) ^a^		0.546 (±0.050) ^c^	
			7	0.482 (±0.048) ^a,b^		0.519 (±0.067) ^a^		0.521 (±0.056) ^a,b^		0.542 (±0.069) ^c^	
**SZAMPION**	**Firmness (N)**	1	1	59.35 (±6.52) ^a^	45.66 *	53.25 (±7.57) ^a^	39.40 *	39.22 (±4.47) ^a^	10.30 *	36.92 (±5.90) ^a^	10.04 *
			7	29.08 (±3.59) ^b,c^		25.94 (±4.41) ^b^		25.91 (±4.08) ^b,c^		21.28 (±3.71) ^c^	
		2	1	31.76 (±5.09) ^b^		29.10 (±2.69) ^b^		26.81 (±2.78) ^b^		28.80 (±3.40) ^b^	
			7	23.80 (±4.42) ^c^		24.01 (±2.42) ^b^		22.57 (±4.82) ^b,c^		21.59 (±3.97) ^c^	
		3	1	31.15 (±4.46) ^b^		28.29 (±4.90) ^b^		25.97 (±3.20) ^b,c^		25.65 (±4.28) ^b,c^	
			7	25.61 (±5.11) ^c^		26.86 (±7.60) ^b^		21.93 (±4.17) ^c^		21.25 (±5.13) ^c^	
	**Biospeckle activity BA**	1	1	0.609 (±0.099) ^a,b^	5.67 *	0.686 (±0.055) ^a,b^	0.19	0.740 (±0.033) ^a^	19.94 *	0.704 (±0.062) ^a^	10.42 *
			7	0.621 (±0.063) ^b^		0.679 (±0.059) ^a,b^		0.606 (±0.038) ^c^		0.621 (±0.056) ^b^	
		2	1	0.622 (±0.058) ^b^		0.675 (±0.060) ^b^		0.639 (±0.059) ^b^		0.653 (±0.046) ^a,b^	
			7	0.594 (±0.071) ^b^		0.656 (±0.082) ^a,b^		0.626 (±0.070) ^b,c^		0.682 (±0.101) ^a^	
		3	1	0.625 (±0.058) ^b^		0.649 (±0.045) ^a,b^		0.618 (±0.058) ^b,c^		0.657 (±0.045) ^a,b^	
			7	0.557 (±0.060) ^a^		0.635 (±0.087) ^a^		0.623 (±0.072) ^b,c^		0.666 (±0.081) ^a,b^	

^a, b, c, d^ Letter indexes indicate membership to homogeneous groups (according to ANOVA with *p* = 0.05). The same superscript letter denotes no significant differences; * means the effect is significant at *p* < 0.05.

**Table 3:** Pearson’s correlation coefficient matrix between maturity indices and biospeckle activity (BA) evaluated for both cultivars separately. * means correlation is significant at *p* < 0.05.

		BA	F	SI	SSC	Streif Index
**Ligol**	**BA**	1.00				
	**F**	−0.53 *	1.00			
	**SI**	0.43 *	−0.67 *	1.00		
	**SSC**	0.63 *	−0.92 *	0.70 *	1.00	
	**Streif Index**	−0.55 *	0.94 *	−0.79 *	−0.95 *	1.00
**Szampion**	**BA**	1.00				
	**F**	−0.51 *	1.00			
	**SI**	0.44 *	−0.67 *	1.00		
	**SSC**	0.40 *	−0.85 *	0.89 *	1.00	
	**Streif Index**	−0.47 *	0.91 *	−0.80 *	−0.95 *	1.00
